# Actionability classification of variants of unknown significance correlates with functional effect

**DOI:** 10.1038/s41698-023-00420-w

**Published:** 2023-07-15

**Authors:** Amber Johnson, Patrick Kwok-Shing Ng, Michael Kahle, Julia Castillo, Bianca Amador, Yujia Wang, Jia Zeng, Vijaykumar Holla, Thuy Vu, Fei Su, Sun-Hee Kim, Tara Conway, Xianli Jiang, Ken Chen, Kenna R. Mills Shaw, Timothy A. Yap, Jordi Rodon, Gordon B. Mills, Funda Meric-Bernstam

**Affiliations:** 1 Institute for Personalized Cancer Therapy, The University of Texas MD Anderson Cancer Center, Houston, TX USA; 2 Bioinformatics and Computational Biology, The University of Texas MD Anderson Cancer Center, Houston, TX USA; 3 Investigational Cancer Therapeutics (Phase I Program), The University of Texas MD Anderson Cancer Center, Houston, TX USA; 4 Division of Oncological Sciences, Knight Cancer Institute, Oregon Health and Science University, Portland, OR USA

**Keywords:** Tumour biomarkers, Clinical genetics

## Abstract

Genomically-informed therapy requires consideration of the functional impact of genomic alterations on protein expression and/or function. However, a substantial number of variants are of unknown significance (VUS). The MD Anderson Precision Oncology Decision Support (PODS) team developed an actionability classification scheme that categorizes VUS as either “Unknown” or “Potentially” actionable based on their location within functional domains and/or proximity to known oncogenic variants. We then compared PODS VUS actionability classification with results from a functional genomics platform consisting of mutant generation and cell viability assays. 106 (24%) of 438 VUS in 20 actionable genes were classified as oncogenic in functional assays. Variants categorized by PODS as Potentially actionable (*N* = 204) were more likely to be oncogenic than those categorized as Unknown (*N* = 230) (37% vs 13%, *p* = 4.08e-09). Our results demonstrate that rule-based actionability classification of VUS can identify patients more likely to have actionable variants for consideration with genomically-matched therapy.

## Introduction

Genomic sequencing is often performed in patients with advanced or metastatic disease in order to identify alterations that may affect therapeutic decision-making and provide additional approved or investigational options. However, not all patients have alterations in actionable genes, and furthermore, not all alterations in actionable genes affect gene function. Current standards and guidelines for delivering tumor genomic sequencing reports within a clinical setting include the requirement for interpretation and categorization of detected variants for their clinical significance^1^. A joint consensus recommendation by the Association for Molecular Pathology, American Society of Clinical Oncology, and College of American Pathologists recommended a four-tiered system designating variants of strong (tier 1), potential (tier 2), unknown (tier 3), and benign (tier 4) clinical significance^2^. Likewise, the FDA recently published a fact sheet detailing three levels of evidence for tumor biomarkers detected within next-generation sequencing tests: companion diagnostic (level 1), clinical significance (level 2), and potential clinical significance (level 3)^3^, and the European Society for Medical Oncology (ESMO) has published the ESMO Scale of Clinical Actionability for molecular Targets (ESCAT)^4^, along with data showing a correlation between improved response and the ESCAT tier^4,5^. Several academic groups have also published their own schemes for determining the level of evidence for targeting a specific genomic alteration with a particular therapy within an indicated tumor type^4,6–20^, and multiple knowledgebases exist to serve as a source of alteration-level interpretation data (e.g., PersonalizedCancerTherapy.org, OncoKB, The Jackson Laboratory Clinical Knowledgebase, as previously reviewed^21^). While these knowledgebases provide essential information for identifying alterations of significance, a large percentage of alterations detected in patient samples have not been previously experimentally or clinically characterized, may not appear within these knowledgebases, and fall within the unknown or uncertain classification (variants of unknown significance, VUS).

In a previous study, we quantified the number of VUS within therapeutically actionable genes identified in patients’ genomic sequencing reports reviewed by the PODS team^22^. 48% of variant annotations provided to oncologists indicated that the variant is a VUS. The large percentage of VUS identified within patients’ sequencing reports presents a great challenge to clinicians. Thus, many groups have developed high-throughput pipelines to characterize functionally somatic and/or germline VUS^23–30^, including a functional genomics platform established at MD Anderson^31^. This platform utilizes two cell lines, MCF10A and Ba/F3, to measure an alteration’s impact on cell viability under growth factor independent conditions. While these platforms generate invaluable information with regards to the clinical actionability of a specific variant, a bottleneck still exists in testing and generating these data in a timely enough manner for point-of-care decision making.

**Fig. 1:**
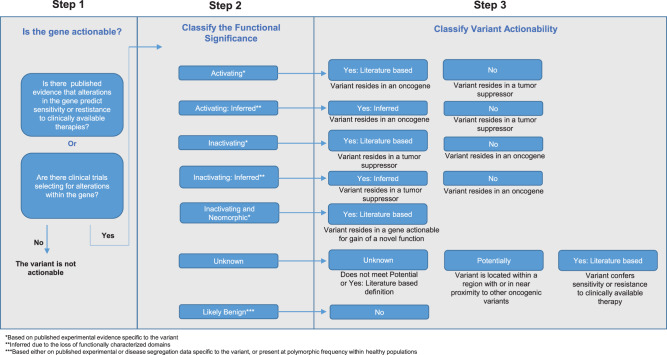
Variant Actionability classification. Schematic depicting PODS’ process of defining variant actionability.

To address the real-time need for determining whether a variant is likely to be functionally significant and therapeutically actionable, the PODS team created a tiered actionability scheme^32^ (Fig. 1). The first step in the scheme is to determine if the gene harboring the variant is therapeutically actionable. PODS scientists classify genes as therapeutically actionable if there is at minimum preclinical evidence that alterations within the gene predict sensitivity or resistance to a clinically available therapy (FDA-approved or investigational agent available within clinical trials) or if alterations within the gene are part of current clinical trial eligibility criteria. Variants within actionable genes are then researched for any known or predicted (inferred) functional impact or therapeutic relevance. Based on these data, PODS assigns a Functional Significance value, which is then utilized to determine Variant Actionability, which may be captured as Yes (based on published literature, inferred due to the loss of characterized domains, or based on functional genomics testing), Potentially, Unknown, or No. For example, activating mutations in the oncogene *BRAF* and inactivating mutations in the tumor suppressor gene *BRCA1* are considered actionable (Supplementary Table 1). If the variant’s Functional Significance is Unknown and there is no known effect of the variant on therapeutic sensitivity or resistance, the variant is classified as either Unknown or Potentially for its Variant Actionability. Variants are Potentially actionable if they are located within a functional domain where other oncogenic variants are known to occur, or, if in general, are within close proximity to other oncogenic variants. Otherwise, these variants are categorized as Unknown for actionability.

In this study, we sought to determine how often VUS in actionable genes are oncogenic using a functional genomics platform that utilizes two cell lines, MCF10A and BaF3, to measure an alteration’s impact on cell viability under growth factor independent conditions^31^. We then determined whether our actionability framework that further classifies VUS into Potentially actionable or Unknown actionability enriches the knowledgebase with variants more likely to be functionally significant, and thus, of therapeutic relevance.

## Results

### Functional characterization of VUS identified by PODS

**Fig. 2:**
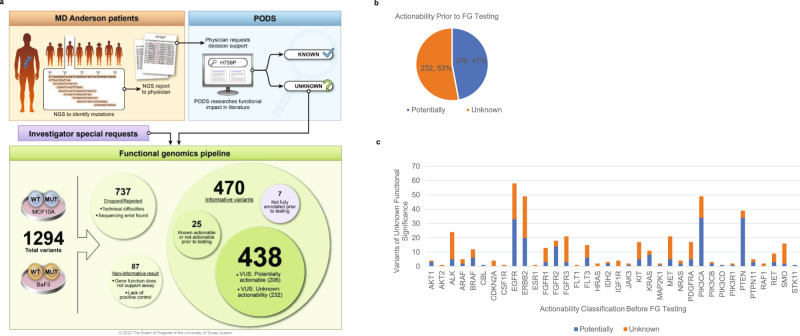
Functional genomics screening of VUS. 1294 variants requested for functional genomics (FG) testing during the years 2015–2019 by the PODS team entered the pipeline for construction of mutant-expressing clones. 737 variants were either dropped from the pipeline due to technical difficulties or subsequently rejected due to a sequencing error found upon validation. Informative results for ingestion within the PODS knowledgebase were obtained for 470 variants. Of these, 438 were fully annotated prior to the functional genomics testing and determined to be variants of unknown functional significance classified as either Unknown (*n* = 232) or Potentially actionable (*n* = 206). Results were excluded for 87 variants deemed non-informative due to the lack of an informative positive control or the function of the gene did not support actionability assessment within the assay. Copyright used with the permission of The Board of Regents of the University of Texas System through The University of Texas MD Anderson Cancer Center (**a**). Percentage of 438 VUS pre-classified as either Unknown or Potentially actionable (**b**) and represented per gene (**c**).

Informative results were obtained for 470 variants requested for functional genomics testing. 438 of these variants (Fig. 2a) were annotated prior to functional genomics testing as Unknown for Functional Significance and either Potentially (*N* = 206, 47%) or Unknown (*N* = 232, 53%) for Variant Actionability (Fig. 2b). The remaining 32 variants were either not annotated prior to testing (*N* = 7) or they were already known to be actionable or not actionable based on data curated from the published literature (*N* = 25). For all 438 variants of unknown functional significance, variants also Unknown for actionability spanned 33 of 36 genes tested, whereas those Potentially actionable spanned 28 of 36 genes tested (Fig. 2c).

**Fig. 3:**
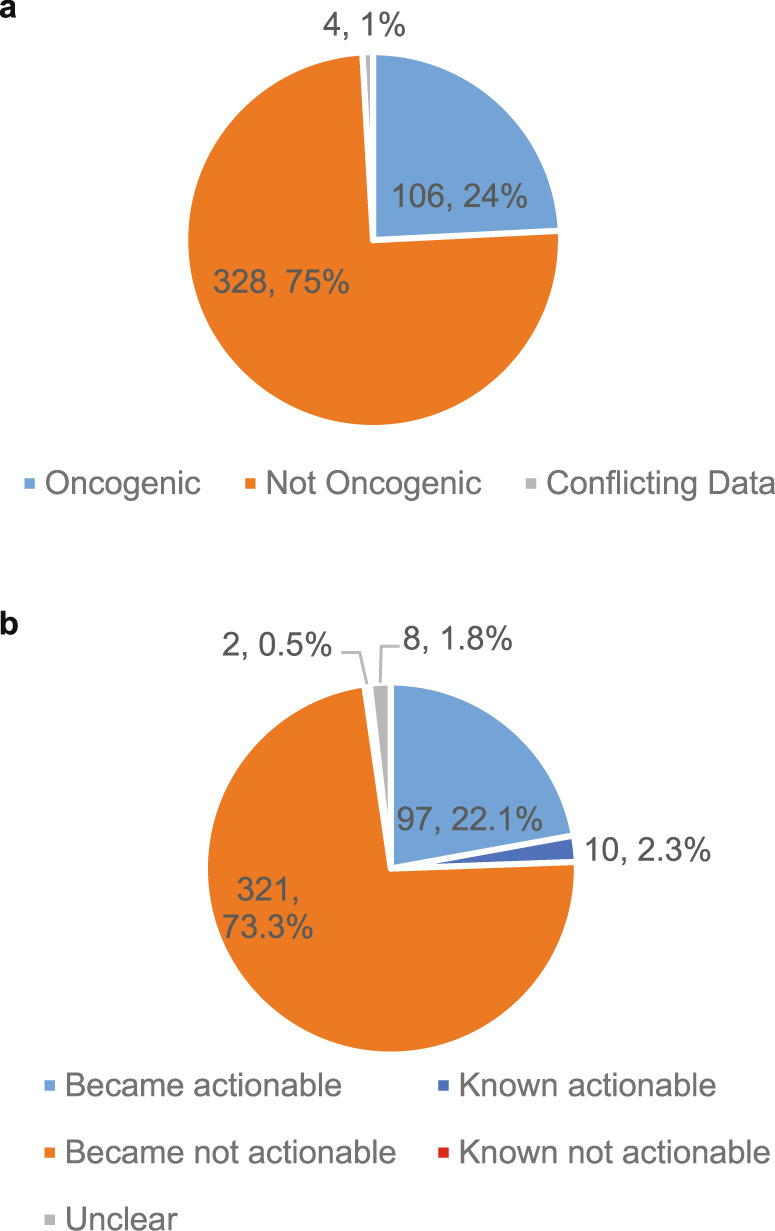
Actionable variants identified from functional genomics screening. Variants of unknown significance were categorized as oncogenic or not oncogenic dependent upon whether the mutation increased cell viability in comparison with expression of the wildtype gene in at least one tested cell line (oncogenic) or either decreased cell viability or conferred no change in cell viability in comparison with the wildtype (not oncogenic). Variants that displayed opposing effects (increased and decreased) in the two cell lines were categorized as conflicting data (**a**). Knowledge accumulated from the published literature within the PODS knowledgebase at the time of functional genomics results were returned was assessed. Variants where literature-based actionability already existed are “Known actionable”, variants where literature-based data existed reflecting a non-actionable assertion are “Known not actionable”, and those that became actionable or not actionable due solely to functional genomics data are shown. If conflicting data existed, the variants are classified as “Unclear” (**b**).

Of the 438 VUS, 106 (24%) increased cell viability in at least one cell line in comparison with its wildtype counterpart (oncogenic), 328 (75%) had no effect differing from wildtype in either cell line or decreased cell viability in comparison with the wildtype (not oncogenic), and 4 (1%) had opposing effects within the two cell lines (conflicting data) (Fig. 3a). After VUS were submitted to the functional genomics platform, new literature was found before functional genomics testing was completed for 12 variants, including 10 (2.3%) variants known to be actionable and 2 (0.5%) known to be not actionable due to newly curated literature (Fig. 3b). Of the 10 known to be actionable variants (Supplementary Table 2), 7 demonstrated a gain-of-function within the published literature that was also observed within the platform. The remaining three variants had no published functional data but were nonetheless considered actionable by PODS due to drug sensitivity or resistance data. The two variants known to be not actionable showed no effect within the functional genomics platform. Eight variants could not be clearly classified as actionable or not actionable due to either cell type-dependent functional effects (4 variants, Fig. 3a) or conflicting data between functional genomics results and the published literature (4 variants, Supplementary Table 3). Thus, 97 (22.1%) variants became actionable and 321 (73.3%) became not actionable due solely to functional genomics data (Fig. 3b).

### PODS classification of VUS as potentially actionable correlates with functional characterization

**Fig. 4:**
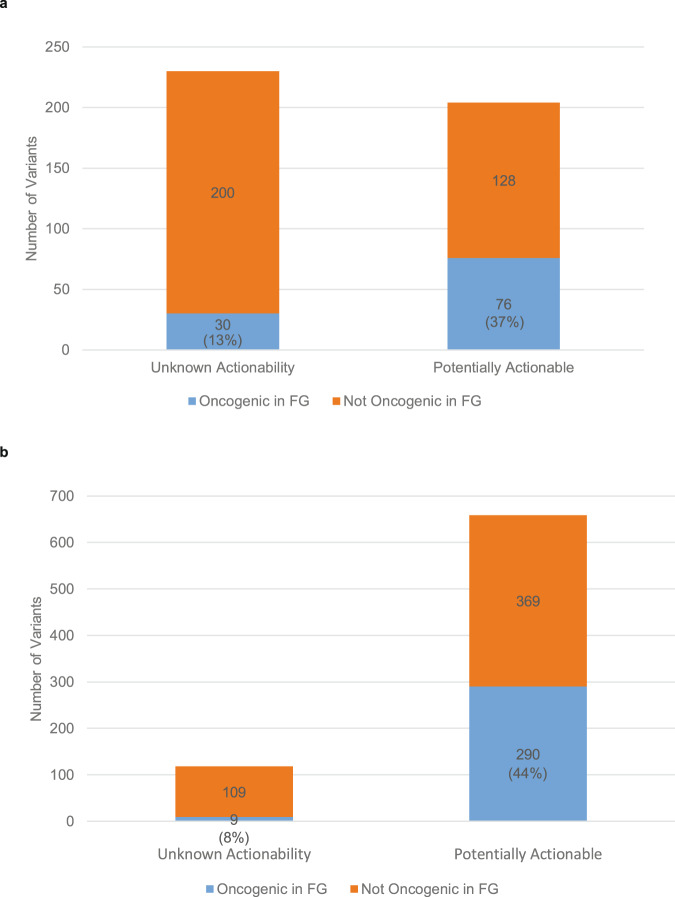
Potentially actionable variants are more likely to be functionally validated. Variants pre-classified as either Unknown or Potentially actionable prior to functional genomics (FG) testing are displayed by whether they were oncogenic (increased cell viability) or not oncogenic (no change or decreased cell viability) within the FG pipeline (Fisher’s Exact Test, odds ratio: 3.94, *p* = 4.08e-09) (**a**). A second set of variants computationally determined to be Potentially actionable or Unknown for actionability are displayed by whether they were oncogenic (increased cell viability) or not oncogenic (no change or decreased cell viability) within the FG pipeline (Fisher’s Exact Test, odds ratio: 9.50, *p* = 4.719e-16) (**b**).

Next, we determined if those variants categorized as Potentially actionable by PODS classification were more likely to be confirmed actionable by functional genomics testing. The four variants that had opposing effects in the two cell lines tested were not included further. 30/230 (13%) variants categorized as Unknown for actionability prior to testing were found to be functionally oncogenic in at least one of the two cell lines tested; whereas, 76/204 (37%) variants categorized as Potentially actionable prior to testing were found to be functionally oncogenic within the functional genomics platform (Fig. 4a). Thus, those annotated as Potentially actionable are more likely to be functionally validated as actionable (Fisher’s Exact Test, odds ratio: 3.94, *p* = 4.08e-09) than those classified as Unknown for actionability. We also computationally applied the PODS Unknown/Potentially actionability classification scheme to a second collection of variants with informative functional genomics results whose testing originates from submission to the platform by other groups (not PODS) or from our team but without prior annotation. Of 777 variants, 6590 were categorized as Potentially actionable based solely upon the described PODS criteria, while 118 were categorized as Unknown. 44% (*n* = 290) of the Potentially actionable variants were verified to be oncogenic in the functional genomics platform compared with only 8% (*n* = 9) of those categorized as Unknown (Fisher’s Exact Test, odds ratio: 9.50, *p* = 4.719e-16; Fig. 4b). Thus, these data support our conclusion that variants annotated by PODS as Potentially actionable are more likely to be oncogenic than those annotated as Unknown for actionability.

**Fig. 5:**
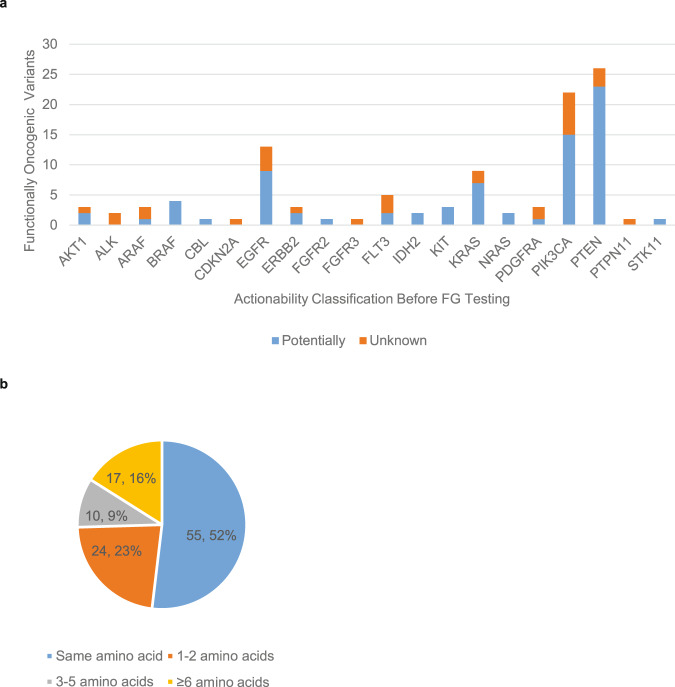
Functionally validated oncogenic variants are in near proximity to other oncogenic variants. Variants pre-classified as either Unknown or Potentially actionable prior to functional genomics (FG) testing and demonstrated to be oncogenic within the FG platform are shown per gene (**a**). The proximity to other known actionable alterations, as determined by accessing the PODS knowledgebase on 9/17/2021, is shown (**b**).

VUS that were oncogenic within the functional genomics platform (*N* = 106, Supplementary Table 4) pre-categorized as Unknown for actionability spanned 13 genes, and those pre-categorized as Potentially actionable spanned 16 genes (Fig. 5a). We next determined proximity for the nearest actionable alteration for all 106 variants determined to be functionally oncogenic in the functional genomics platform and pre-categorized as either Unknown or Potentially actionable. Two variants were excluded as they are truncating mutations, which are typically assigned an actionability value based on what is known in the published literature regarding the functional impact of the lost protein region and not solely on its proximity to other known actionable alterations. Of those 104 variants examined, there is an actionable alteration at the same amino acid position or within the amino acid span (for in-frame insertions and/or deletions) for 52% of the variants (Fig. 5b). For another 23% of variants, another actionable alteration exists within at least 2 amino acids. Thus, the majority of VUS demonstrated to be oncogenic within the functional genomics platform are within 2 amino acids of another alteration also demonstrated to be actionable based on the published literature or functional genomics testing.

## Discussion

Somatic genomic sequencing is recommended for all patients when one or more genomic biomarkers are linked to a regulatory body-approved therapy in the patient’s tumor type^33^. These genomic alterations are designated as AMP Tier 1A^1^/ PODS level 1A^34^ and have the highest level of evidence for clinical action. If the drug approval is linked to a specific alteration, such as *BRAF* V600E, interpreting the results and therapeutic choices is relatively straight forward. However, some FDA indications and professional guidelines are linked to a general type of alteration and not a specific variant. For example, erdafitinib is FDA approved for the treatment of urothelial cancers with susceptible *FGFR3* or *FGFR2* genetic alterations, per FDA label^35^. In this case, functionally characterizing novel *FGFR2* and *FGFR3* alterations has significant therapeutic implications. For patients where no Tier 1A alterations are detected or they were previously acted upon, identifying functionally significant alterations that may be predictive of response to targeted therapies investigated within the context of a clinical trial becomes equally as important. However, a large portion of cancer-associated mutations have not been functionally characterized. Among all 16,738 annotated alterations within PODS as of 4/27/2022, 65% are not known for their therapeutic actionability (Unknown or Potentially actionable, Supplementary Fig. 1). Moreover, in a previous study, we determined that approximately 50% of 535 patients assessed by the PODS team had no clearly actionable mutation to pursue for enrollment on a clinical trial at the time of assessment^22^, and a similar study found only 41% of patient samples had a potentially actionable mutation^10^.

The ideal scenario for determining whether a VUS is likely to be actionable is to experimentally test the function of the alteration. Functional genomics platforms are one way to characterize the tumorigenic potential of a large number of mutations. The platform utilized in this study measures a mutation’s impact on cell viability in growth factor independent conditions compared with expression of its wildtype counterpart. 24% of mutations tested increased cell viability (Fig. 3a), providing evidence that they may be tumor-promoting events that could potentially confer sensitivity to targeted therapies. For the remainder of the variants, these may either be benign passenger mutations, or their tumorigenic properties may not be seen in the genetic background of the cell models used and/or the assay setting of the platform. For example, we observed several mutations within *FGFR2* that confer increased survival in the presence of FGF ligand, but not in its absence (data not shown). Additionally, we acknowledge another limitation of the current platform. Some variants may promote other tumorigenic phenotypes such as migration or angiogenesis, which are not assessed on this platform. Therefore, we excluded variants (i.e., annotated as non-informative) residing in genes where neither the wildtype not any variation of the gene promoted cell viability to avoid over interpretation of the testing result.

While the functional analysis data are value-adding for variant-level knowledgebases, such as PODS, the information is typically not generated quick enough to influence care for the initial patient for which the alteration was identified. Notably, the PODS functional genomics effort was initiated with the intent to be able to guide decision-making for individual patients. However, as more of the advanced cancer population underwent comprehensive testing on platforms that go beyond “hot spot” testing for recurrent mutations, it quickly became apparent that the current genomics platform does not have fast enough turn-around to guide the care of individual cancer patients who often have rapidly progressing disease. Therefore, instead, we embarked on systematic characterization of recurrent VUS in known drivers, in order to impact subsequent patients with these mutations. In the future, tracking functional impact of individual mutations shared from larger scale functional genomics efforts, as well as tracking individual clinical outcome data of patients with genomic alterations treated on genomically-informed trials, will likely improve decision support efforts.

Methods for predicting the likelihood that an alteration is tumor-promoting are value-adding when functional data is not available. Multiple informatics tools, such as Mutation Assessor^36^, Hotspot3D^37^, HotMAPS^38^, SIFT^39^, Polyphen-2^40^, FATHMM-XF^41^, CanPredict^42^, MutationTaster^43^, SNAP^44^, GAVIN^45^, EVE^46^, CGI^47,48^, VEST4^49^, CScape^50^, and CHASM^51^ were developed for this purpose. These tools use a variety of properties and features to predict the functional impact of a mutation, including evolutionary conservation, protein features, 3D protein structures, machine learning from curated driver mutations, and other codon-specific physiochemical properties. Some tools such as CanDrA^52^ combine features across tools to make a prediction. Another tool, e-MutPath, assesses the effect mutations have on functional pathways by overlapping gene expression perturbations in cancer with patient-specific mutations and identified perturbations in protein-protein interactions^53^. With so many tools and options that may give varying predictions, it can be difficult to discern the best approach, although various comparisons have been made^31,52,54,55^. We chose four widely used prediction tools in order to assess how they perform relative to our functional genomics results (Supplementary Fig. 2). Alterations predicted to be drivers by CGI^47^ (30% vs 10%; Fisher’s Exact Test, odds ratio: 4.08, *p* = 3.221e-06), VEST4^49^ (35% vs 12%; Fisher’s Exact Test, odds ratio: 3.99, *p* = 4.187e-08), CHASMplus^56^ (30% vs 6%; Fisher’s Exact Test, odds ratio: 6.23, *p* = 9.849e-09), and CScape^50^ (24% vs 4%; Fisher’s Exact Test, odds ratio: 7.71, *p* = 0.01449) were more likely to be oncogenic in the functional genomics platform than those predicted by the respective tools to be passengers. However, with large-scale decision support efforts, the only known input may be the amino acid change, limiting the application of some bioinformatic prediction tools. Indeed, not all alterations could be called by each tool. With only protein amino acid change as input, 360/434 (83%) were called by CGI, 401/434 (92%) were called by VEST4, 401/434 (92%) were called by CHASMplus, and 373/434 (86%) were called by CScape. Thus, the PODS team’s schema is complimentary to these tools. It relies on curated knowledge of known functional mutations likely to be oncogenic in conjunction with manual assessment of protein domains to classify a VUS as Potentially actionable if it resides within the same functional domain as other oncogenic mutations and/or is located in close proximity or at the same codon as other oncogenic mutations. This approach is supported by literature demonstrating that functionally significant, non-frameshift/truncating alterations tend to cluster in specific functional regions of the gene. For example, 17/20 of the most frequent *PIK3CA* mutations in breast cancer^57^ that are also oncogenic, reside within a region characterized and captured within UniProt^58^.

Additionally, if an uncharacterized mutation is located at a hotspot, defined as a recurrently mutated amino acid in cancer, it may be considered more likely to be pathogenic. Hot spot annotation databases can be useful for predicting functional effect^59^. For example, the hotspot *KRAS* codon G12 is substituted for a variety of other amino acids within cancer samples. Many of these substitutions have been shown to be oncogenic and/or confer the same functional effect of impairing hydrolysis of GTP, (A^60,61^/C^61,62^/D^61^/F^63^/R^61^/S^60,64^/V^61^/Y^65^), albeit to differing degrees. Thus, other non-characterized variants of G12 would be considered Potentially actionable. However, other recurrently mutated codons are polymorphisms and benign in nature, such as *KIT* M541L (rs3822214, dbSNP). Thus, the PODS team does not rely on frequency of detection to differentiate between Potentially actionable and Unknown for actionability variants. Our approach necessitates that other alterations at the hotpot or functionally characterized region alter protein function in a manner that is likely tumor promoting in order for VUS at that codon or region to be classified as Potentially actionable.

Until this study, the merit of our tiered actionability scheme for VUS had not been tested for the value of a Potential call. Our data here show that alterations categorized as Potentially actionable by the criteria described are more likely to be functionally significant than those categorized as Unknown for actionability (Fig. 4), as demonstrated in cell viability assays. We also demonstrate that the majority of the functionally validated variants are in near proximity (1–2 amino acids) to other oncogenic variants (Fig. 5b). These data suggest that among the Potentially actionable variants, there may be even more stratification of likelihood that is useful: those within 2 amino acids of another oncogenic variant being even more likely to be functionally oncogenic.

There has been some debate about how to optimize efficacy signal in genomically-informed trials. In our study, of the 438 VUS, only 24% were oncogenic. This supports the idea that when genomically-informed trials are conducted, if the goal is to enhance the efficacy signal, accrual either should be limited to known alterations, or alternately incorporate a functional annotation step that will incorporate emerging alterations with literature support and provide a tiered classification of VUS for consideration of enrollment in selected scenarios.

Altogether these data demonstrate that functional annotations relying on experimental data cannot be replaced by predicted functionality by proximity and protein features, as 63% of VUS classified as Potentially actionable were not functionally validated in the systems assessed (Fig. 4). However, the PODS tiered VUS actionability scheme does add value in stratifying alterations more likely to be functionally significant: 37% of the Potentially actionable variants had a functionally significant effect in the functional genomics platform. This information would be important to take into consideration for an individual patient along with expected therapeutic efficacy of the genomically-matched therapy and other treatment options available. Therefore, genomic annotation of VUS may identify additional patients that benefit from emerging therapeutics.

## Methods

### Clinical genomic testing and PODS variant annotations

Patients underwent genomic testing using local or commercial clinical genomic next-generation sequencing tests as standard of care or under genomic sequencing studies with written informed consent (NCT01772771). The prospective genomic testing protocol (with written informed consent), as well as a protocol for retrospective review of clinical genomic testing results (with waiver of informed consent) was reviewed and approved by the MD Anderson Cancer Center Institutional Review Board. Variants identified within patients’ CLIA sequencing reports are entered into the PODS knowledgebase. PODS scientists classified genes as therapeutically actionable (Fig. 1)^32^. Variants within actionable genes were then researched for any known or predicted functional impact or therapeutic relevance. Based on these data, PODS assigned a Functional Significance classification, which was then utilized to determine the Variant Actionability classification. Some variants may have more than one Variant Actionability value; each value associated with treatment or resistance to a specific drug or class of drugs (Supplementary Table 1). In these cases, the highest value was utilized for all analyses within the paper (Yes > Potentially > Unknown > No).

We also computationally applied our rules for assignment of an Unknown or Potentially actionable value to a second set of variants, which originated from other groups also utilizing the functional genomics platform or variants submitted by PODS without prior annotation (Fig. 4b). Like with manual annotation, a Potentially actionable value is given if either the alteration resides within a protein feature considered functional (disordered regions excluded) and that contains at least one actionable mutation of the subtype missense, in-frame insertion, in-frame deletion, duplication, or deletion-insertion within or overlapping with the amino acid range of the alteration, or the alteration is within five amino acids of an actionable alteration of the subtypes previously specified irrespective of location within a functionally characterized protein feature.

### Variant submission and testing

1,294 variants requested for testing by the PODS team during the years 2015–2019 entered the functional genomics pipeline (Fig. 2a). Lentiviral vectors, originating from Clontech, expressing variants of interest or corresponding wildtype were constructed and validated as previously described^29^. Seven hundred thirty-seven variants were dropped out during the process due to various technical reasons, including unavailability of correct ORF and failure in full-length sequencing validation. Expression vectors for 557 variants were constructed, full-length sequencing validated, and functional testing proceeded with two growth factor-dependent cell line models, Ba/F3 and MCF10A, as previously described^31^. Briefly, lentivirus vectors expressing either the wildtype gene or the variant of interest were expressed within the two cell lines. Ba/F3 cells originate from MD Anderson Characterized Cell Line Core facility, and MCF10A cells originate from ATCC (CRL-10317). Transduced cells were incubated without dependent growth factors (i.e. IL-3 for Ba/F3, EGF and insulin for MCF10A) for 3 weeks. Cell viability was measured during the 3-week assay period, and the effect of the variant was compared with the corresponding wild type. Results were considered informative (470 variants) for utilization within the PODS knowledgebase if expression of at least one variant of the gene or the wildtype gene promoted cell viability within the cell line; thus, demonstrating that the oncogenic potential of the gene can be observed in the genetic background of the cell model utilized. Otherwise, results were considered non-informative in the respective cell line(s). 87 variant results were deemed non-informative for this reason or because the wildtype gene functioned in a manner opposite of the effect being examined for actionability. Specifically, expression of *FGF6*, typically considered an oncogene^66,67^, suppressed cell growth in the assay; and *PTCH1*, typically considered a tumor suppressor gene^68,69^, increased cell viability. Thus, we could not confidently assess mutations for actionable gain-of-function (*FGF6*) or loss-of-function (*PTCH1*) mutations. *ARAF* mutations were only considered non-informative within MCF10A cells, as the wildtype gene demonstrated tumor suppressive activity within this cell line but not Ba/F3 cells. *ARAF* is typically considered an oncogene^70,71^; thus, oncogenic gain-of-function mutations within MCF10A cells could not be determined.

### Determining functionally validated, actionable variants

Variants were considered actionable by functional genomics testing if they increased cell survival and/or proliferation in comparison with the wildtype gene in at least one cell line tested, and the variant resides within a gene classified as actionable by the PODS team at the time the result was captured. At the time of data capture, four variants remained Unknown for Functional Significance after functional genomics testing after considering their effect within the platform in combination with what was known at the time within the published literature. Detailed annotations are provided in Supplementary Table 3 for these variants. A two-sided Fisher’s Exact Test was performed to determine if those annotated as potentially actionable were more likely to be functionally validated as actionable.

### Utilizing bioinformatic prediction tools

The publicly available web interface for the Cancer Genome Interpreter^47^, available at https://www.cancergenomeinterpreter.org, was utilized to categorize variants as a driver (predicted and/or annotated) or a passenger. Additionally, OpenCravat^72^ was utilized to access prediction tools VEST4^73^, CHASMplus^56^, and CScape^50^. First, protein amino acid changes were mapped to a genomic position and DNA change via TransVar^74^ version 2.5.10.20211024 using UCSC reference genome HG19 that was indexed by samtools (version 1.17)^75^. Outputs from TransVar were checked against input protein changes, and incorrect mappings were removed. For variants with multiple inferences, results were retained for those where the cDNA and protein amino acid change match between TransVar and OpenCravat. OpenCravat inferred variants with the most severe sequence ontology as primary variants and retained other mapped transcripts as secondary information. Alterations, where the inferred amino acid change from the prediction tool does not match with the amino acid change serving as input from functional genomics testing, were discarded from inclusion. For inferences with primary transcripts or protein changes that did not match between Transvar and OpenCravat, we selected the MANE and Ensembl transcripts with the same protein changes for CHASMplus and VEST4. As no transcript information for CScape was available in OpenCravat, we excluded predictions for those unmatched from the analysis. A significance level of 0.05 was used for determining predicted oncogenic drivers versus passengers for CHASMplus and VEST4, and a threshold of 0.5 was used for CScape as recommended by the tool.

### Determining the nearest oncogenic variant

To determine the nearest oncogenic variant to a “variant of interest” that was validated to be actionable by functional genomics testing, a search of the PODS database was conducted on 9/17/2021. Variants that had at least one actionability value of “Yes” based on published literature or functional genomics testing and are of the subtype missense, in-frame deletion, in-frame insertion, duplication, or deletion-insertion qualified as “other” oncogenic variants. The distance between the two variants was calculated as follows:

- When the variant of interest represents a single codon and the other oncogenic variant represents a single codon, the difference between the two codons was subtracted (e.g, D323A and D323E; distance = 0)
- When the variant of interest represents a single codon and the other oncogenic variant comprises multiple codons, the distance was determined to be 0 if the variant of interest (e.g, K385M) resides within the amino acid range of the other oncogenic variant (e.g, Y375_K455del)
- When the variant of interest comprises multiple codons and the other oncogenic variant represents a single codon, the distance was calculated to be 0 if the other oncogenic variant (e.g., Y65C) resides within the range of amino acids for the variant of interest (e.g., H64_Y65_delinsQS).
- When the variant of interest comprises multiple codons and the other oncogenic variant also comprises multiple codons, the distance was calculated as 0 if the amino acid range of the two variants are identical or the amino acid range of either the variant of interest or the nearest oncogenic variant is nested within the other’s amino acid range (e.g., P551_M552 > L and K550_K558del). For all other scenarios, the distance is calculated as the difference between the two most N-terminal amino acids (e.g., D770_N771insGF and N771_P772insH, distance = 1)

### Reporting summary

Further information on research design is available in the Nature Research Reporting Summary linked to this article.

## Supplementary information

REPORTING SUMMARY

Supplementary Material

## Data Availability

Patients’ tumors were sequenced within a very large variety of external CLIA-certified laboratories, in addition to MD Anderson’s internal CLIA-certified laboratory. Clinical sequencing data was collected and entered within an internal MD Anderson database as part of the informed consent protocol (NCT01772771) from these various sources. The PODS team accessed sequencing data within the MD Anderson database and determined variants of unknown significance, upon physician request. The mutations identified and tested within the functional genomics platform for all variants referenced within the paper are available at https://ibl.mdanderson.org/fasmic/#!/. The accession number is FASMIC00230421.

## Code availability

Code is available for generation of bioinformatic tool-predicted oncogenicity values (Supplementary Fig. 2) at https://github.com/KChen-lab/Data-Analysis-of-Variant-Functional-Effects.
