# Supplementary material for: Actionability classification of variants of unknown significance correlates with functional effect
Source: NPJ Precis Oncol. 2023 Jul 15;7:67. doi: 10.1038/s41698-023-00420-w (PMC10349825; doi:10.1038/s41698-023-00420-w)
Supplement: Supplementary file 1 — REPORTING SUMMARY [file 41698_2023_420_MOESM1_ESM.pdf]

nature portfolio

Corresponding author(s): Funda Meric-Bernstam

Last updated by author(s): Apr 26, 2023

Reporting Summary

Nature Portfolio wishes to improve the reproducibility of the work that we publish. This form provides structure for consistency and transparency in reporting. For further information on Nature Portfolio policies, see our Editorial Policies and the Editorial Policy Checklist.

Statistics

For all statistical analyses, confirm that the following items are present in the figure legend, table legend, main text, or Methods section.

n/a	Confirmed	
☐	☒	The exact sample size (*n*) for each experimental group/condition, given as a discrete number and unit of measurement
☐	☒	A statement on whether measurements were taken from distinct samples or whether the same sample was measured repeatedly
☐	☒	The statistical test(s) used AND whether they are one- or two-sided<br>*Only common tests should be described solely by name; describe more complex techniques in the Methods section.*
☐	☒	A description of all covariates tested
☐	☒	A description of any assumptions or corrections, such as tests of normality and adjustment for multiple comparisons
☐	☒	A full description of the statistical parameters including central tendency (e.g. means) or other basic estimates (e.g. regression coefficient) AND variation (e.g. standard deviation) or associated estimates of uncertainty (e.g. confidence intervals)
☐	☒	For null hypothesis testing, the test statistic (e.g. *F*, *t*, *r*) with confidence intervals, effect sizes, degrees of freedom and *P* value noted<br>*Give P values as exact values whenever suitable.*
☒	☐	For Bayesian analysis, information on the choice of priors and Markov chain Monte Carlo settings
☒	☐	For hierarchical and complex designs, identification of the appropriate level for tests and full reporting of outcomes
☐	☒	Estimates of effect sizes (e.g. Cohen's *d*, Pearson's *r*), indicating how they were calculated

Our web collection on statistics for biologists contains articles on many of the points above.

Software and code

Policy information about availability of computer code

Data collection

1) Functional genomics data were collected directly from the laboratory. These data were then compared with predicted oncogenicity categories from CGI, VEST4, CHASMplus, and CScape as described in the data analysis section below. 2) A projected actionability call was computationally generated for alterations described in Figure 4b based on manually curated knowledge within MD Anderson's Precision Oncology Knowledgebase.

Data analysis

1) Preprocessing and postprocessing was performed in R (version 4.2.2) on x86_64-apple-darwin17.0 (64-bit) [1]. Reverse annotation was performed using Transvar (version 2.5.10.20211024) [2] with UCSC reference genome HG19 [3] indexed by samtools (version 1.17) [4]. Variant annotations were performed using web interface of OpenCravat (version 2.3.0) [5] to obtain predicted oncogenic driver classifications from CHASMplus (version 1.3.0) [6], VEST4 (version 4.4.0) [7,8], and CScape (version 1.0.1) [9].

[1] R Core Team (2022). R: A language and environment for statistical computing. R
Foundation for Statistical Computing, Vienna, Austria. URL https://www.R-project.org/.

[2] Zhou et al. Nature Methods 12, 1002-1003 (2015). http://www.nature.com/nmeth/journal/v12/n11/full/nmeth.3622.html

[3] UCSC Genome Browser. "Golden Path hg19 (GRCh37) Assembly." Available at: ftp://hgdownload.cse.ucsc.edu/goldenPath/hg19/chromosomes/. Accessed March 20, 2023.

[4] Li, H., & Durbin, R. (2009). Fast and accurate short read alignment with Burrows-Wheeler transform. Bioinformatics, 25(14), 1754-1760. (SAMtools version 1.17)

[5] Pagel KA et al. Integrated Informatics Analysis of Cancer-Related Variants. JCO Clinical Cancer Informatics 2020 4, 310-317.

[6] Tokheim C, Karchin R. CHASMplus Reveals the Scope of Somatic Missense Mutations Driving Human Cancers. Cell Syst. 2019 Jul 24;9(1):9-23.e8. doi: 10.1016/j.cels.2019.05.005. Epub 2019 Jun 12. PMID: 31202631; PMCID: PMC6857794.

[7] Carter H, Douville C, Yeo G, Stenson PD, Cooper DN, Karchin R (2013) Identifying Mendelian disease genes with the Variant Effect Scoring Tool BMC Genomics. 14(3) 1-16.

[8] Douville C, Masica DL, Stenson PD, Cooper DN, Gygax D, Kim R, Ryan M, Karchin R (2016) Assessing the pathogenicity of insertion and deletion variants with the Variant Effect Scoring Tool (VEST-indel) Human Mutation 37(1):28-35.

[9] Rogers MF, Shihab H, Gaunt TR, Campbell C (2017). CScape: a tool for predicting oncogenic single-point mutations in the cancer genome. Nature Scientific Reports

2) For each gene of interest, a set of mutations called qualifying alterations (QA) are determined, which are then cross referenced with the curated protein domains within PODS, flagging those that have an overlap as qualifying domains (QD). QAs are those alterations manually annotated within the PODS knowledgebase to have an oncogenic functional effect. To produce a projected actionability call for a given alteration, if it overlaps with any QD, or it is within the vicinity of other QAs, then a Potentially call will be given; otherwise, a Unknown call will be predicted. Nested domains have been considered and the qualifying status of the most inner domain that overlaps with the alteration of interest is used in the final determination.

For manuscripts utilizing custom algorithms or software that are central to the research but not yet described in published literature, software must be made available to editors and reviewers. We strongly encourage code deposition in a community repository (e.g. GitHub). See the Nature Portfolio guidelines for submitting code & software for further information.

Data

Policy information about availability of data

All manuscripts must include a data availability statement. This statement should provide the following information, where applicable:
- Accession codes, unique identifiers, or web links for publicly available datasets
- A description of any restrictions on data availability
- For clinical datasets or third party data, please ensure that the statement adheres to our policy

The functional genomics data is available at https://ibl.mdanderson.org/fasmic/#!/. The accession numbers is FASMIC00230421. Code is available for generation of bioinformatic tool predicted oncogenicity values (Supplemental Figure 2) at https://github.com/KChen-lab/Data-Analysis-of-Variant-Functional-Effects.

Research involving human participants, their data, or biological material

Policy information about studies with human participants or human data. See also policy information about sex, gender (identity/presentation), and sexual orientation and race, ethnicity and racism.

Reporting on sex and gender	There is no reporting based on sex and gender. While genomic alterations were originally identified from patient's genomic sequencing results, they were then annotated for their actionability in a patient-agnostic manner. No patient-specific variables were considered within this manuscript.
Reporting on race, ethnicity, or other socially relevant groupings	There is no reporting on race, ethnicity, or other socially relevant grouping.
Population characteristics	There are no population characteristics described within the manuscript.
Recruitment	Patients were not specifically recruited for this study. Instead, the manuscript relies on genomic sequencing data collected by the institution at large under informed consent protocol NCT01772771.
Ethics oversight	MD Anderson Cancer Center Institutional Review Board

Note that full information on the approval of the study protocol must also be provided in the manuscript.

Field-specific reporting

Please select the one below that is the best fit for your research. If you are not sure, read the appropriate sections before making your selection.

☒ Life sciences ☐ Behavioural & social sciences ☐ Ecological, evolutionary & environmental sciences

For a reference copy of the document with all sections, see nature.com/documents/nr-reporting-summary-flat.pdf

Life sciences study design

All studies must disclose on these points even when the disclosure is negative.

Sample size	All functional genomics results and PODS annotations meeting the defined criteria in the manuscript were included.

Data exclusions	Data exclusions are described in the methods and briefly restated here. Functional genomics results were considered non-informative and not included in the paper if neither the wildtype nor any variation tested of it increased cell viability, or if the wildtype behaved opposite of the actionable functional effect. Specifically mutations in FGF6, ARAF (MCF10A cells only), and PTCH1 were excluded for this reason.
Replication	Replication of the functional genomics results themselves is described within the primary paper reporting these results: PMID: 29533785.
Randomization	Randomization is not applicable to the study design.
Blinding	Blinding is not applicable to the study design.

Reporting for specific materials, systems and methods

We require information from authors about some types of materials, experimental systems and methods used in many studies. Here, indicate whether each material, system or method listed is relevant to your study. If you are not sure if a list item applies to your research, read the appropriate section before selecting a response.

Materials & experimental systems

n/a	Involved in the study	
☒	☐	Antibodies
☐	☒	Eukaryotic cell lines
☒	☐	Palaeontology and archaeology
☒	☐	Animals and other organisms
☒	☐	Clinical data
☒	☐	Dual use research of concern
☒	☐	Plants

Methods

n/a	Involved in the study	
☒	☐	ChIP-seq
☒	☐	Flow cytometry
☒	☐	MRI-based neuroimaging

Eukaryotic cell lines

Policy information about cell lines and Sex and Gender in Research

Cell line source(s)	Ba/F3 cells originate from M.D. Anderson Characterized Cell Line Core facility (Houston, TX). MCF10A cells originate from ATCC (CRL-10317). LetntiX-293T cells originated from Clontech (Cat#632180).
Authentication	Ba/F3 cells were validated based on continued dependence on IL-3 for propagation (mouse originated cell line). MCF10A cells were authenticated by Short Tandem Repeat (STR) analysis at M.D. Anderson Characterized Cell Line Core facility (Houston, TX)
Mycoplasma contamination	Cell lines were negative for mycoplasma.
Commonly misidentified lines (See ICLAC register)	These are not commonly misidentified cell lines.
