# Supplementary material for: Actionability classification of variants of unknown significance correlates with functional effect
Source: NPJ Precis Oncol. 2023 Jul 15;7:67. doi: 10.1038/s41698-023-00420-w (PMC10349825; doi:10.1038/s41698-023-00420-w)
Supplement: Supplementary file 2 — Supplementary Material [file 41698_2023_420_MOESM2_ESM.pdf]

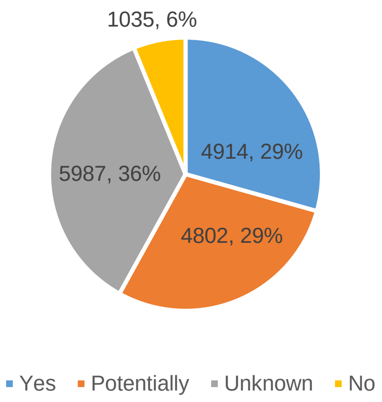

Supplementary Figure 1. The percentage of fully annotated alterations within the PODS knowledgebase as of 4/27/2022 that are categorized as Yes (Yes: Literature based, Yes: Inferred, Yes: Functional Genomics), Potentially, Unknown, or No (No, No: Functional Genomics) for variant actionability for treatment with or resistance to clinically available matched therapies or enrollment on clinical trials.

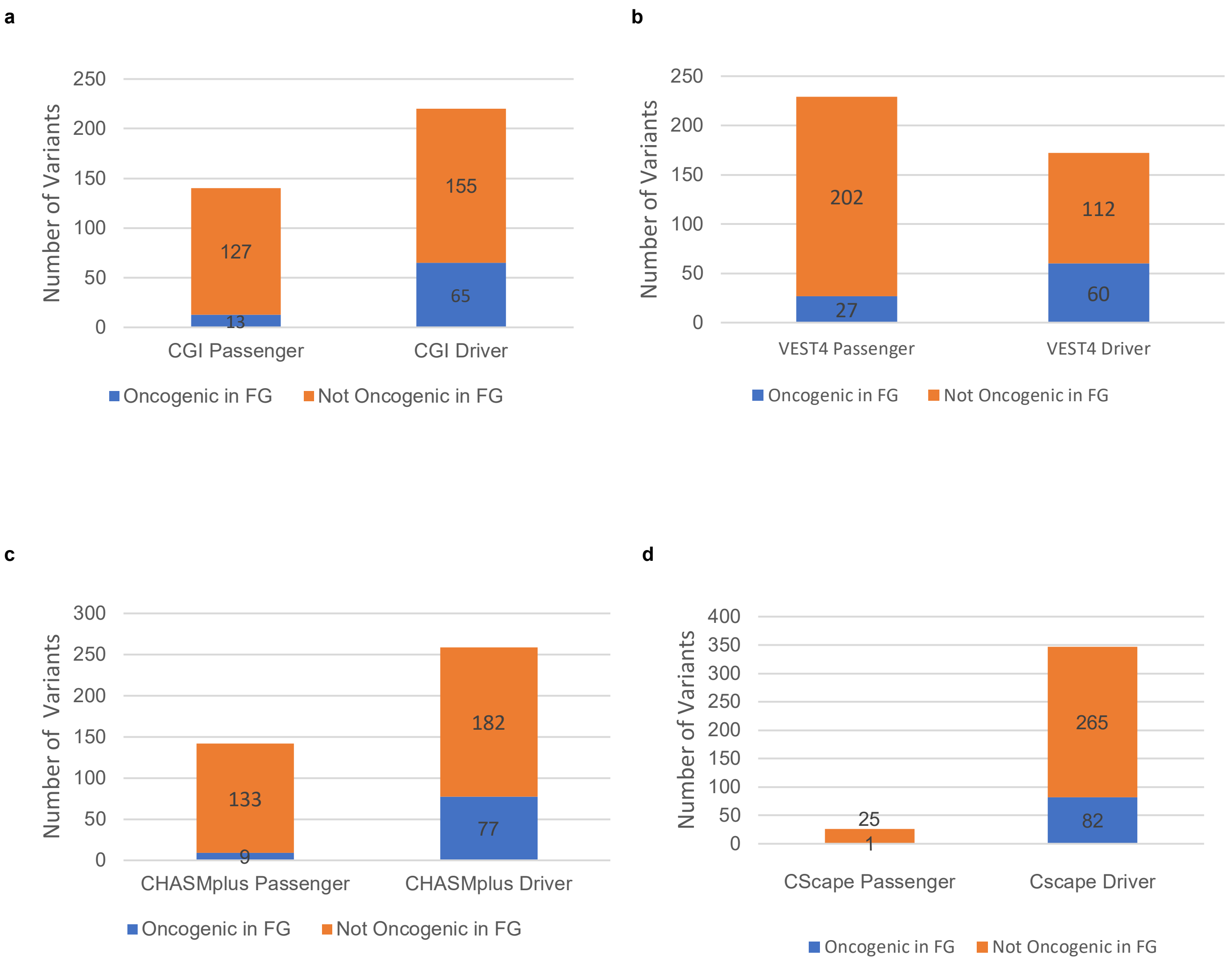

Supplementary Figure 2. Comparison of driver/passenger classification by informatic prediction tools with functional genomics results. Alterations classified by CGI (a), VEST4 (b), CHASMplus (c), or CScape (d) as either passengers or drivers are shown relative to their classification within the functional genomics platform as oncogenic or not oncogenic.

Supplementary Table 1. Examples of PODS Annotations and Classification of Variant Actionability

Variant	Annotation	Functional Significance	Variant Actionability
**BRAF V600E**	*BRAF* V600E is the most commonly reported *BRAF* alteration. V600E occurs within the BRAF kinase domain (amino acids 457-717; UniProt). This alteration gains 200- to 500-fold activity over the wild-type BRAF, constitutively activates MEK/ERK, resulting in up-regulation of cyclin D1 in the absence of extracellular signal, and induces cellular transformation[1-5]. A genomic alteration resulting in this amino acid change is recorded within dbSNP (rs113488022, rs121913377). The clinical significance of this alteration in ClinVar[6] is pathogenic, drug response, other, likely pathogenic (VCV000013961.74;June, 2022).	Activating	Yes: Literature based (for treatment with BRAF, MEK or ERK inhibitors)
**NTRK3 G623R**	The functional effect of this alteration on NTRK3 kinase activity has not been experimentally determined. It is located within the kinase domain of the protein (amino acids 538-839, UniProt[7]). Gain-of-function missense mutations have been reported in this region[8]. However, we are currently unaware of any experimental data demonstrating an increase in kinase activity with this specific mutation. *NTRK3* G623R confers resistance to FDA approved Trk inhibitors larotrectinib and entrectinib. *NTRK3* 623R is referred to as a solvent front mutation that confers resistance by sterically hindering drug binding[9-11]. This alteration is not reported in dbSNP or ClinVar[6] databases (June, 2022).	Unknown	Yes:Literature based (for resistance to larotrectinib or entrectinib within the context of a susceptible *NTRK3* fusion)
**AKT1 D323E**	This alteration is considered activating. This alteration is located within the kinase domain (amino acids 150-408, UniProt[7]), which is essential for the function of AKT1[12,13]. Expression of *AKT1* D323E in BaF3 and MCF10A cells resulted in moderately increased cell viability and/or growth compared with expression of the wildtype gene in the Institute of Personalized Cancer Therapeutics Functional Genomics laboratory (Unpublished Observations). There is currently no published data detailing the functional consequence of this alteration. This alteration is not reported in dbSNP or ClinVar[6] (June, 2022) databases.	Activating	Yes: Functional Genomics (for treatment with AKT or mTOR inhibitors)
**BRCA1 A1708E**	This alteration has been reported to be inactivating. A1708E is also called c.5123C>A and is reported to be a founder *BRCA1* mutation in Hispanic populations of breast and/or ovarian cancer[14,15]. Causality analysis, which takes into consideration that this variant segregates primarily with diseased populations and rarely co-occurs with other deleterious *BRCA1* variants, shows A1708E favors causality for cancer risk[16-18]. This pathogenicity is confirmed by multiple functional studies that show A1708E strongly disrupts BRCA1 stability and activity[19-27]. Furthermore, c.5123C>A has been shown to alter *BRCA1* exon 18 splicing showing partial in-frame skipping of exon 18, which encodes a portion of the BRCT domain, which is either the primary mechanism for A1708E's loss-of-function effect or it contributes to *BRCA1* inactivation[28]. A genomic alteration resulting in this amino acid change is recorded within dbSNP (rs28897696). The clinical significance of this alteration in ClinVar[6] is pathogenic (VCV000055407.31; VCV000868230.2June, 2022).	Inactivating	Yes: Literature based (for treatment with PARP inhibitors and platinum-based chemotherapy)

Supplementary Table 1. Examples of PODS Annotations and Classification of Variant Actionability

**NOTCH1 S2426fs*81**	Although the functional consequence of this alteration has not been experimentally tested, it is inferred to be activating. *NOTCH1* S2426fs*81 creates a frameshift at amino acid 2426 and subsequent premature truncation 8 amino acids downstream within the cytoplasmic PEST domain (amino acids 2424-2555) of Notch1 protein (UniProt[7]). The PEST domain is a negative regulatory sequence of *NOTCH1* , and truncating mutations within this domain have been reported to increase the Notch intracellular domain (NICD) half-life and thus activate NOTCH1 protein[29,30]. This alteration is not reported in dbSNP or ClinVar[6] (June, 2022) databases.	Activating: Inferred	Yes: Inferred (for treatment with gamma secretase inhibitors)
**BRCA1 K223fs*11**	Although this alteration has not been experimentally tested, it is inferred to be inactivating. This alteration has not been reported in dbSNP or ClinVar[6] (June, 2022) databases. This alteration results in a shift in the coding frame of *BRCA1* at codon 223, and premature protein truncation 11 amino acids downstream. K223fs*11 introduces a truncation within the region that interacts with the SWI/SNF chromatin remodeling complex subunit BRD7 (amino acids 1-300[31]), which facilitates the recruitment of BRCA1 to the promoter of specific genes, such as *ER alpha* . This event eliminates translation of the vast majority of the 1863 amino acid protein, including the essential BRCT1/2 (1642-1736, 1756-1855) and SCD (1280-1524) domains and the regions that interact with FANCA, Rad51 (740-1083), and PALB2 (1397-1424) (UniProt[7]). BRCT1/2 domains interact with various cell cycle checkpoints, repair proteins, chromatin remodeling, and transcription factors[32,33]; SCD is important for BRCA1-mediated checkpoint activation; PALB2-BRCA1 interaction serves as the molecular scaffold[33,34], which are all essential to the tumor suppressor function of BRCA1. Collectively, this alteration is predicted to result in loss-of-function due to disruption and/or elimination of essential protein domains.	Inactivating: Inferred	Yes: Inferred (for treatment with PARP inhibitors and platinum-based chemotherapy)
**ERBB2 G727A**	This alteration has not been functionally characterized. G727A alteration occurs within the kinase domain (amino acids 720-987) of *ERBB2* (UniProt[7]). Mutations within this region of *ERBB2* have been previously reported in tumors and are highly activating mutations[35-37]. Thus, there is the potential that this alteration confers a similar effect; however, it has not been experimentally determined. This alteration is not reported in dbSNP or ClinVar[6] databases (June, 2022).	Unknown	Potentially (for treatment with HER2 inhibitors)
**ERBB2 Q646H**	This alteration is of unknown functional significance. It is not located within a functionally characterized domain (UniProt[7]). This alteration is not reported in dbSNP or ClinVar[6] databases (May, 2022).	Unknown	Unknown (for treatment with HER2 inhibitors)
**BRCA1 V191I**	This alteration was characterized to result in neutral effect on *BRCA1* function. V191I is located close to the ring finger domain[38]. Functional studies demonstrated that this alteration resulted in comparable function to the wild type in homologous directed recombination, proliferation, and cisplatin sensitivity assays[25,39]. A genomic alteration resulting in this amino acid change is recorded within dbSNP (rs80357090). The clinical significance of this alteration in ClinVar[6] is Benign (VCV000037684.49; June, 2022).	Likely Benign	No (not actionable for treatment with PARP inhibitors and platinum-based chemotherapy)

Supplementary Table 1. Examples of PODS Annotations and Classification of Variant Actionability

**ERBB2 Q156***	Although this alteration has not been functionally characterized, it is inferred to be inactivating. Q156* occurs within the domain I (amino acid 52-173, UniProt[7]) and would eliminate the kinase domain of ERBB2 protein (amino acids 720-987, UniProt[7]). Following the dimerization of ERBB2, the kinase domain is activated and regulates downstream cell proliferation and survival (MAPK and PI3K) pathways[40]. An *in vitro* model suggested that the lack of kinase domain interrupted ERBB2 dimerization and activation process[41]. Therefore, this alteration is predicted to be inactivating. This alteration is not reported in dbSNP or ClinVar[6] databases (June, 2022).	Inactivating: Inferred	No (not actionable for treatment with HER2 inhibitors)
**AKT1 A58V**	This alteration is of likely benign functional significance. Expression of this alteration in BaF3 and MCF10A cells resulted in no change in cell viability and/or growth compared to the wildtype in the IPCT Functional Genomics laboratory. A58V occurs at a conserved residue and is located within the N-terminal pleckstrin homology (PH) domain[7,42], which recruits AKT1 to the plasma membrane and is required for AKT1 activation[43]. A genomic alteration leading to this amino acid change is recorded in dbSNP (rs1892948595, May, 2022). It is not reported within ClinVar[6] (June, 2022).	Likely Benign	No: Functional Genomics (not actionable for treatment with AKT or mTOR inhibitors)

Supplementary Table 2. Variants known to be actionable at receipt of functional genomics testing results

Variant	Functional genomics effect	Known Literature-based Functional effect	Known Literature-based Variant Actionability	Final PODS Functional Significance	Final PODS Variant Actionability
**ALK_G1202R**	NDFW	Unknown	Yes: Literature based (for resistance to crizotinib, ceritinib, alectinib, and brigatinib in the context of an ALK fusion; for treatment with lorlatinib in the context of an ALK fusion)[44-52]	Unknown	Yes: Literature based (for resistance to crizotinib, ceritinib, alectinib, and brigatinib in the context of an ALK fusion; for treatment with lorlatinib in the context of an ALK fusion)
**EGFR_E709_T710 delinsD**	Activating	Activating	Yes: Literature based (for treatment with EGFR inhibitors)[53]	Activating	Yes: Literature based (for treatment with EGFR inhibitors)
**EGFR_N771_H773 dupNPH**	Activating	Activating: Inferred	Yes: Inferred (for resistance to first and second generation EGFR inhibitors, for treatment with poziotinib or pan-ERBB inhibitors)[54-63]	Activating	Yes: Inferred (for resistance to first and second generation EGFR inhibitors, for treatment with poziotinib or pan-ERBB inhibitors)
**EGFR_V441G**	Inactivating	Unknown	Yes: Literature based (for resistance to cetuximab and panitumumab)[64]	Inactivating	Yes: Literature based (for resistance to cetuximab and panitumumab)
**EGFR_V769M**	Activating	Activating	Yes: Literature based (for treatment with EGFR inhibitors)[65]	Activating	Yes: Literature based (for treatment with EGFR inhibitors)
**ERBB2_D277Y**	NDFW	Unknown	Yes: Literature based (for treatment with HER2 inhibitors)[66]	Unknown	Yes: Literature based (for treatment with HER2 inhibitors)
**IDH2_R172W**	Activating	Inactivating and Neomorphic	Yes: Literature Based (for treatment with IDH inhibitors)[67-69]	Inactivating and Neomorphic	Yes: Literature Based (for treatment with IDH inhibitors)
**KRAS_G13P**	Activating	Activating	Yes: Literature based (for resistance to cetuximab, panitumumab, erlotinib, and gefitinib; for treatment with MEK inhibitors)[70-77]	Activating	Yes: Literature based (for resistance to cetuximab, panitumumab, erlotinib, and gefitinib; for treatment with MEK inhibitors)
**PIK3CA_I112N**	Activating	Activating	Yes: Literature based (for treatment with PI3K, AKT, or mTOR inhibitors)[78]	Activating	Yes: Literature based (for treatment with PI3K, AKT, or mTOR inhibitors)
**PIK3CA_Q546E**	Activating	Activating	Yes: Literature based (for treatment with PI3K, AKT, or mTOR inhibitors)[5,79]	Activating	Yes: Literature based (for treatment with PI3K, AKT, or mTOR inhibitors)

Supplementary Table 3. Variants that remained classified as a VUS after functional genomics testing due to consideration of the published literature.

Gene	Alteration	BaF3 Call	MCF10A Call	Consolidated FG-based Call	Final PODS Functional Significance Call	Final PODS Variant Actionability Call	PODS Annotation
CBL	Y371N	Activating	non-informative	Activating	Unknown	Potentially	Y371, as well as F700 and F774, is phosphorylated by the insulin receptor kinase in response to insulin. Y371 is located within the linker region (amino acids 352-380, UniProt[7]) between the SH2 and Ring finger domains[80]. Expression of *CBL* Y371N within BaF3 cells led to an increase in cellular proliferation and/or viability within the IPCT Functional Genomics laboratory when compared with expression of the wildtype gene. *CBL* is typically considered a tumor suppressor[81]; however, expression of the wildtype *CBL* gene had no effect when compared with expression of a negative control. Thus, it is unclear by what mechanism expression of the mutation led to increased cellular proliferation and/or viability. One hypothesis is that the mutant serves as a dominant negative inhibiting the function of the endogenous CBL protein, but this has not been experimentally tested. While the functional effect of this mutation has not been tested within the published literature, Y371F was investigated. This mutation impaired CBL-mediated EGFR ubiquitination and degradation and diminished the loss of phosphorylation seen with mutation of Y700 and Y774[82].
PTEN	R173L	Not Different From Wildtype	Not Different From Wildtype	Not Different From Wildtype	Unknown	Potentially	*PTEN* R173L alteration has not been functionally characterized within the published literature. R173L alteration is located within the phosphatase tensin-type domain of PTEN protein (UniProt[7]). Other alterations at the same codon are known to be inactivating, R173C[83-85] and R173H[83,86]. However, expression of this variant in BaF3 and MCF10A cells resulted in no change in cell viability and/or growth compared with expression of the wildtype gene in the IPCT Functional Genomics laboratory. Given that other variants at this codon confer a loss-of-function and the mutation is classified as likely pathogenic in ClinVar[6] (VCV000428258.5), it is possible that this alteration does effect phosphatase activity but does not confer a change in cell survival in this specific assay.
PTEN	T167I	Not Different From Wildtype	Not Different From Wildtype	Not Different From Wildtype	Unknown	Potentially	This alteration is of unknown functional significance within the published literature. T167I is located within the phosphatase tensin-type domain of PTEN, which mediates the dephosphorylation of phosphatidylinositol 3,4,5-trisphosphate (PIP3) and inhibits PI3K signaling. Disruption of this domain has been shown to impair PTEN phosphatase activity and promote PI3K, AKT and MTOR signaling[86]. Other cancer- and disease-associated mutations located within this region, have been shown to be inactivating and tumor promoting mutations[87-89]. In fact, another alteration of the same codon, T167A, was experimentally characterized to decreases PTEN phosphatase activity[86]. However, expression of this variant in BaF3 and MCF10A cells resulted in no change in cell viability and/or growth compared with expression of the wildtype gene in the IPCT Functional Genomics laboratory. T167I has been previously reported in endometrial cancer[90]. Given that other variants at the same codon confer a loss-of-function, it is possible that this alteration does effect phosphatase activity but does not confer a change in cell survival in this specific assay.
STK11	P314H	Activating	Activating	Activating	Unknown	Potentially	This mutation is of unknown functional significance within the published literature. It has been reported in a patient with Peutz-Jeghers syndrome, a rare autosomal-dominant disorder characterized by the formation of benign hamartomatous polyps in the gastrointestinal tract[91]. Germline *STK11* mutations are found in approximately 70% of patients with near complete penetrance in patients harboring mutations in the gene[91]. This alteration was also detected as a somatic mutation in a patient with colorectal cancer[92]. P314 is located just C-terminal to the kinase domain (amino acids 49-309, UniProt[7]), but not within a functionally characterized region. Expression of *STK11* P314H in BaF3 and MCF10A cells resulted in a weak increase in cell viability and/or growth compared with expression of the wildtype gene in the IPCT Functional Genomics laboratory. *STK11* is generally considered a tumor suppressor gene[93]; however, expression of the wildtype gene had no effect on cellular proliferation or survival. Thus, it is unclear by what mechanism expression of the mutation led to increased cellular proliferation and/or viability. There is currently no published data detailing the functional consequence of this alteration. The clinical significance of this alteration in ClinVar[6] is uncertain significance (VCV001327848.1; Jan, 2022).

Supplementary Table 4. Variants classified as actionable based on functional genomics testing

AKT1_D323E
AKT1_D323N
AKT1_N53H
ALK_C443DEL
ALK_Q27L
ARAF_E195A
ARAF_S214P
ARAF_V218I
BRAF_L597H
BRAF_N581H
BRAF_T241M
BRAF_V600DUP
CBL_Y371N
CDKN2A_L78fs*41
EGFR_A289P
EGFR_C620F
EGFR_D1009E
EGFR_D770_N771insGF
EGFR_E709_T710delinsD
EGFR_N1116D
EGFR_N771_H773dupNPH
EGFR_N771_P772insH
EGFR_Q217K
EGFR_Q432R
EGFR_R222C
EGFR_T751_I759delinsN
EGFR_V769M
ERBB2_L841V
ERBB2_P551L
ERBB2_Y590C
FGFR2_F276C
FGFR3_R124W
FLT3_A450V
FLT3_D586H
FLT3_Q577H
FLT3_R437K
FLT3_S584F
IDH2_R140L
IDH2_R172W
KIT_N566D
KIT_P551_M552>L
KIT_W557_E562DEL
KRAS_A146P
KRAS_A59T

Supplementary Table 4. Variants classified as actionable based on functional genomics testing

KRAS_G13P
KRAS_G13S
KRAS_G60S
KRAS_N116H
KRAS_R68S
KRAS_S65G
KRAS_S65I
NRAS_G12A
NRAS_G13V
PDGFRA_E556K
PDGFRA_K385M
PDGFRA_Y288C
PIK3CA_C378F
PIK3CA_D1017H
PIK3CA_D1029H
PIK3CA_D725K
PIK3CA_E110del
PIK3CA_E418K
PIK3CA_E542V
PIK3CA_G1007D
PIK3CA_H450_V461DELINSGS
PIK3CA_I1058L
PIK3CA_I112N
PIK3CA_I910V
PIK3CA_K111_I112delinsN
PIK3CA_L456R
PIK3CA_L658F
PIK3CA_Q546E
PIK3CA_Q546L
PIK3CA_Q75E
PIK3CA_R108C
PIK3CA_R93P
PIK3CA_Y1021C
PIK3CA_Y1021H
PTEN_A126T
PTEN_C136F
PTEN_D24G
PTEN_D331fs*13
PTEN_G127R
PTEN_G132D
PTEN_G132S
PTEN_H272P
PTEN_H64_Y65_DELINSQS
PTEN_I122N

Supplementary Table 4. Variants classified as actionable based on functional genomics testing

PTEN_I253N
PTEN_I28T
PTEN_L100P
PTEN_L247_P248del
PTEN_L25V
PTEN_L325R
PTEN_L57S
PTEN_L70H
PTEN_L70V
PTEN_M134DEL
PTEN_N12T
PTEN_N48D
PTEN_T26N
PTEN_V119F
PTEN_W111R
PTEN_Y27C
PTPN11_S502P
STK11_P314H

References for Supplementary Tables

1. Cantwell-Dorris, E. R., O'Leary, J. J. & Sheils, O. M. BRAFV600E: implications for carcinogenesis and molecular therapy. *Mol Cancer Ther* **10**, 385-394, doi:10.1158/1535-7163.MCT-10-0799 (2011).
2. Namba, H. *et al.* Clinical implication of hot spot BRAF mutation, V599E, in papillary thyroid cancers. *J Clin Endocrinol Metab* **88**, 4393-4397, doi:10.1210/jc.2003-030305 (2003).
3. Wan, P. T. *et al.* Mechanism of activation of the RAF-ERK signaling pathway by oncogenic mutations of B-RAF. *Cell* **116**, 855-867, doi:10.1016/s0092-8674(04)00215-6 (2004).
4. Houben, R. *et al.* Constitutive activation of the Ras-Raf signaling pathway in metastatic melanoma is associated with poor prognosis. *J Carcinog* **3**, 6, doi:10.1186/1477-3163-3-6 (2004).
5. Ng, P. K. *et al.* Systematic Functional Annotation of Somatic Mutations in Cancer. *Cancer Cell* **33**, 450-462 e410, doi:10.1016/j.ccell.2018.01.021 (2018).
6. Landrum, M. J. *et al.* ClinVar: improving access to variant interpretations and supporting evidence. *Nucleic Acids Res* **46**, D1062-D1067, doi:10.1093/nar/gkx1153 (2018).
7. UniProt, C. UniProt: the universal protein knowledgebase in 2021. *Nucleic Acids Res* **49**, D480-D489, doi:10.1093/nar/gkaa1100 (2021).
8. Genevois, A. L. *et al.* Dependence receptor TrkC is a putative colon cancer tumor suppressor. *Proc Natl Acad Sci U S A* **110**, 3017-3022, doi:10.1073/pnas.1212333110 (2013).
9. Drilon, A. *et al.* What hides behind the MASC: clinical response and acquired resistance to entrectinib after ETV6-NTRK3 identification in a mammary analogue secretory carcinoma (MASC). *Ann Oncol* **27**, 920-926, doi:10.1093/annonc/mdw042 (2016).
10. Drilon, A. *et al.* Efficacy of Larotrectinib in TRK Fusion-Positive Cancers in Adults and Children. *N Engl J Med* **378**, 731-739, doi:10.1056/NEJMoa1714448 (2018).
11. Liu, D., Offin, M., Harnicar, S., Li, B. T. & Drilon, A. Entrectinib: an orally available, selective tyrosine kinase inhibitor for the treatment of NTRK, ROS1, and ALK fusion-positive solid tumors. *Ther Clin Risk Manag* **14**, 1247-1252, doi:10.2147/TCRM.S147381 (2018).
12. Vanhaesebroeck, B. & Alessi, D. R. The PI3K-PDK1 connection: more than just a road to PKB. *Biochem J* **346 Pt 3**, 561-576 (2000).
13. Nicholson, K. M. & Anderson, N. G. The protein kinase B/Akt signalling pathway in human malignancy. *Cell Signal* **14**, 381-395, doi:10.1016/s0898-6568(01)00271-6 (2002).
14. Ferla, R. *et al.* Founder mutations in BRCA1 and BRCA2 genes. *Ann Oncol* **18 Suppl 6**, vi93-98, doi:10.1093/annonc/mdm234 (2007).

References for Supplementary Tables

15 Torres, D. *et al.* High proportion of BRCA1/2 founder mutations in Hispanic breast/ovarian cancer families from Colombia. *Breast Cancer Res Treat* **103**, 225-232, doi:10.1007/s10549-006-9370-1 (2007).

16 Easton, D. F. *et al.* A systematic genetic assessment of 1,433 sequence variants of unknown clinical significance in the BRCA1 and BRCA2 breast cancer-predisposition genes. *Am J Hum Genet* **81**, 873-883, doi:10.1086/521032 (2007).

17 Lindor, N. M. *et al.* A review of a multifactorial probability-based model for classification of BRCA1 and BRCA2 variants of uncertain significance (VUS). *Hum Mutat* **33**, 8-21, doi:10.1002/humu.21627 (2012).

18 Capanu, M. *et al.* Assessment of rare BRCA1 and BRCA2 variants of unknown significance using hierarchical modeling. *Genet Epidemiol* **35**, 389-397, doi:10.1002/gepi.20587 (2011).

19 Lee, M. S. *et al.* Comprehensive analysis of missense variations in the BRCT domain of BRCA1 by structural and functional assays. *Cancer Res* **70**, 4880-4890, doi:10.1158/0008-5472.CAN-09-4563 (2010).

20 Caligo, M. A., Bonatti, F., Guidugli, L., Aretini, P. & Galli, A. A yeast recombination assay to characterize human BRCA1 missense variants of unknown pathological significance. *Hum Mutat* **30**, 123-133, doi:10.1002/humu.20817 (2009).

21 Vallon-Christersson, J. *et al.* Functional analysis of BRCA1 C-terminal missense mutations identified in breast and ovarian cancer families. *Hum Mol Genet* **10**, 353-360, doi:10.1093/hmg/10.4.353 (2001).

22 Williams, R. S. *et al.* Detection of protein folding defects caused by BRCA1-BRCT truncation and missense mutations. *J Biol Chem* **278**, 53007-53016, doi:10.1074/jbc.M310182200 (2003).

23 Humphrey, J. S. *et al.* Human BRCA1 inhibits growth in yeast: potential use in diagnostic testing. *Proc Natl Acad Sci U S A* **94**, 5820-5825, doi:10.1073/pnas.94.11.5820 (1997).

24 Gaboriau, D. C., Rowling, P. J., Morrison, C. G. & Itzhaki, L. S. Protein stability versus function: effects of destabilizing missense mutations on BRCA1 DNA repair activity. *Biochem J* **466**, 613-624, doi:10.1042/BJ20141077 (2015).

25 Bouwman, P. *et al.* A high-throughput functional complementation assay for classification of BRCA1 missense variants. *Cancer Discov* **3**, 1142-1155, doi:10.1158/2159-8290.CD-13-0094 (2013).

26 Mirkovic, N., Marti-Renom, M. A., Weber, B. L., Sali, A. & Monteiro, A. N. Structure-based assessment of missense mutations in human BRCA1: implications for breast and ovarian cancer predisposition. *Cancer Res* **64**, 3790-3797, doi:10.1158/0008-5472.CAN-03-3009 (2004).

27 Millot, G. A. *et al.* Assessment of human Nter and Cter BRCA1 mutations using growth and localization assays in yeast. *Hum Mutat* **32**, 1470-1480, doi:10.1002/humu.21608 (2011).

References for Supplementary Tables

28 Sanz, D. J. *et al.* A high proportion of DNA variants of BRCA1 and BRCA2 is associated with aberrant splicing in breast/ovarian cancer patients. *Clin Cancer Res* **16**, 1957-1967, doi:10.1158/1078-0432.CCR-09-2564 (2010).

29 Weng, A. P. *et al.* Activating mutations of NOTCH1 in human T cell acute lymphoblastic leukemia. *Science* **306**, 269-271, doi:10.1126/science.1102160 (2004).

30 Aster, J. C. *et al.* Essential roles for ankyrin repeat and transactivation domains in induction of T-cell leukemia by notch1. *Mol Cell Biol* **20**, 7505-7515, doi:10.1128/MCB.20.20.7505-7515.2000 (2000).

31 Harte, M. T. *et al.* BRD7, a subunit of SWI/SNF complexes, binds directly to BRCA1 and regulates BRCA1-dependent transcription. *Cancer Res* **70**, 2538-2547, doi:10.1158/0008-5472.CAN-09-2089 (2010).

32 Anantha, R. W. *et al.* Functional and mutational landscapes of BRCA1 for homology-directed repair and therapy resistance. *Elife* **6**, doi:10.7554/eLife.21350 (2017).

33 Huen, M. S., Sy, S. M. & Chen, J. BRCA1 and its toolbox for the maintenance of genome integrity. *Nat Rev Mol Cell Biol* **11**, 138-148, doi:10.1038/nrm2831 (2010).

34 Sy, S. M., Huen, M. S. & Chen, J. PALB2 is an integral component of the BRCA complex required for homologous recombination repair. *Proc Natl Acad Sci U S A* **106**, 7155-7160, doi:10.1073/pnas.0811159106 (2009).

35 Wang, S. E. *et al.* HER2 kinase domain mutation results in constitutive phosphorylation and activation of HER2 and EGFR and resistance to EGFR tyrosine kinase inhibitors. *Cancer Cell* **10**, 25-38, doi:10.1016/j.ccr.2006.05.023 (2006).

36 Ng, C. K. *et al.* Intra-tumor genetic heterogeneity and alternative driver genetic alterations in breast cancers with heterogeneous HER2 gene amplification. *Genome Biol* **16**, 107, doi:10.1186/s13059-015-0657-6 (2015).

37 Fan, Y. X. *et al.* Mutational activation of ErbB2 reveals a new protein kinase autoinhibition mechanism. *J Biol Chem* **283**, 1588-1596, doi:10.1074/jbc.M708116200 (2008).

38 Chang, J., Hilsenbeck, S. G., Sng, J. H., Wong, J. & Ragu, G. C. Pathological features and BRCA1 mutation screening in premenopausal breast cancer patients. *Clin Cancer Res* **7**, 1739-1742 (2001).

39 Lu, C. *et al.* Patterns and functional implications of rare germline variants across 12 cancer types. *Nat Commun* **6**, 10086, doi:10.1038/ncomms10086 (2015).

40 Herter-Sprie, G. S., Greulich, H. & Wong, K. K. Activating Mutations in ERBB2 and Their Impact on Diagnostics and Treatment. *Front Oncol* **3**, 86, doi:10.3389/fonc.2013.00086 (2013).

References for Supplementary Tables

41 Xu, W. *et al.* Sensitivity of mature Erbb2 to geldanamycin is conferred by its kinase domain and is mediated by the chaperone protein Hsp90. *J Biol Chem* **276**, 3702-3708, doi:10.1074/jbc.M006864200 (2001).

42 Russell, M. A. Synemin Redefined: Multiple Binding Partners Results in Multifunctionality. *Front Cell Dev Biol* **8**, 159, doi:10.3389/fcell.2020.00159 (2020).

43 Mahadevan, D. *et al.* Discovery of a novel class of AKT pleckstrin homology domain inhibitors. *Mol Cancer Ther* **7**, 2621-2632, doi:10.1158/1535-7163.MCT-07-2276 (2008).

44 Katayama, R. *et al.* Mechanisms of acquired crizotinib resistance in ALK-rearranged lung Cancers. *Sci Transl Med* **4**, 120ra117, doi:10.1126/scitranslmed.3003316 (2012).

45 Hallberg, B. & Palmer, R. H. The role of the ALK receptor in cancer biology. *Ann Oncol* **27 Suppl 3**, iii4-iii15, doi:10.1093/annonc/mdw301 (2016).

46 Ignatius Ou, S. H. *et al.* Next-generation sequencing reveals a Novel NSCLC ALK F1174V mutation and confirms ALK G1202R mutation confers high-level resistance to alectinib (CH5424802/RO5424802) in ALK-rearranged NSCLC patients who progressed on crizotinib. *J Thorac Oncol* **9**, 549-553, doi:10.1097/JTO.0000000000000094 (2014).

47 Dagogo-Jack, I. *et al.* Treatment with Next-Generation ALK Inhibitors Fuels Plasma ALK Mutation Diversity. *Clin Cancer Res* **25**, 6662-6670, doi:10.1158/1078-0432.CCR-19-1436 (2019).

48 Lin, Y. T., Yu, C. J., Yang, J. C. & Shih, J. Y. Anaplastic Lymphoma Kinase (ALK) Kinase Domain Mutation Following ALK Inhibitor(s) Failure in Advanced ALK Positive Non-Small-Cell Lung Cancer: Analysis and Literature Review. *Clin Lung Cancer* **17**, e77-e94, doi:10.1016/j.cllc.2016.03.005 (2016).

49 Friboulet, L. *et al.* The ALK inhibitor ceritinib overcomes crizotinib resistance in non-small cell lung cancer. *Cancer Discov* **4**, 662-673, doi:10.1158/2159-8290.CD-13-0846 (2014).

50 Gainor, J. F. *et al.* Molecular Mechanisms of Resistance to First- and Second-Generation ALK Inhibitors in ALK-Rearranged Lung Cancer. *Cancer Discov* **6**, 1118-1133, doi:10.1158/2159-8290.CD-16-0596 (2016).

51 Zou, H. Y. *et al.* PF-06463922, an ALK/ROS1 Inhibitor, Overcomes Resistance to First and Second Generation ALK Inhibitors in Preclinical Models. *Cancer Cell* **28**, 70-81, doi:10.1016/j.ccell.2015.05.010 (2015).

52 Shaw, A. T. *et al.* Lorlatinib in non-small-cell lung cancer with ALK or ROS1 rearrangement: an international, multicentre, open-label, single-arm first-in-man phase 1 trial. *Lancet Oncol* **18**, 1590-1599, doi:10.1016/S1470-2045(17)30680-0 (2017).

References for Supplementary Tables

53 Kobayashi, Y. *et al.* EGFR Exon 18 Mutations in Lung Cancer: Molecular Predictors of Augmented Sensitivity to Afatinib or Neratinib as Compared with First- or Third-Generation TKIs. *Clin Cancer Res* **21**, 5305-5313, doi:10.1158/1078-0432.CCR-15-1046 (2015).

54 Yasuda, H. *et al.* Structural, biochemical, and clinical characterization of epidermal growth factor receptor (EGFR) exon 20 insertion mutations in lung cancer. *Sci Transl Med* **5**, 216ra177, doi:10.1126/scitranslmed.3007205 (2013).

55 Yasuda, H., Kobayashi, S. & Costa, D. B. EGFR exon 20 insertion mutations in non-small-cell lung cancer: preclinical data and clinical implications. *Lancet Oncol* **13**, e23-31, doi:10.1016/S1470-2045(11)70129-2 (2012).

56 Robichaux, J. P. *et al.* Mechanisms and clinical activity of an EGFR and HER2 exon 20-selective kinase inhibitor in non-small cell lung cancer. *Nat Med* **24**, 638-646, doi:10.1038/s41591-018-0007-9 (2018).

57 Hirano, T. *et al.* In vitro modeling to determine mutation specificity of EGFR tyrosine kinase inhibitors against clinically relevant EGFR mutants in non-small-cell lung cancer. *Oncotarget* **6**, 38789-38803, doi:10.18632/oncotarget.5887 (2015).

58 Fang, W. *et al.* EGFR exon 20 insertion mutations and response to osimertinib in non-small-cell lung cancer. *BMC Cancer* **19**, 595, doi:10.1186/s12885-019-5820-0 (2019).

59 Yang, M. *et al.* NSCLC harboring EGFR exon-20 insertions after the regulatory C-helix of kinase domain responds poorly to known EGFR inhibitors. *Int J Cancer* **139**, 171-176, doi:10.1002/ijc.30047 (2016).

60 Yuza, Y. *et al.* Allele-dependent variation in the relative cellular potency of distinct EGFR inhibitors. *Cancer Biol Ther* **6**, 661-667, doi:10.4161/cbt.6.5.4003 (2007).

61 Engelman, J. A. *et al.* PF00299804, an irreversible pan-ERBB inhibitor, is effective in lung cancer models with EGFR and ERBB2 mutations that are resistant to gefitinib. *Cancer Res* **67**, 11924-11932, doi:10.1158/0008-5472.CAN-07-1885 (2007).

62 Harada, T. *et al.* Characterization of epidermal growth factor receptor mutations in non-small-cell lung cancer patients of African-American ancestry. *Oncogene* **30**, 1744-1752, doi:10.1038/onc.2010.545 (2011).

63 Pasi A. Janne, J. W. N., D. Ross Camidge, Alexander I. Spira, Zofia Piotrowska, Leora Horn, Daniel Botelho Costa, Anne S. Tsao, Jyoti D. Patel, Shirish M. Gadgeel, Lyudmila Bazhenova, Viola Weijia Zhu, Howard West, Sylvie Vincent, Jian Zhu, Shuanglian Li, Gregory J. Riely. Antitumor activity of TAK-788 in NSCLC with EGFR exon 20 insertions. *Journal of Clinical Oncology* **37**, suppl 9007-9007, doi:10.1200/JCO.2019.37.15_suppl.9007 (2019).

References for Supplementary Tables

64 Strickler, J. H. *et al.* Genomic Landscape of Cell-Free DNA in Patients with Colorectal Cancer. *Cancer Discov* **8**, 164-173, doi:10.1158/2159-8290.CD-17-1009 (2018).

65 Hellmann, M. D. *et al.* Identification and Functional Characterization of EGFR V769M, a Novel Germline Variant Associated With Multiple Lung Adenocarcinomas. *JCO Precis Oncol* **1**, doi:10.1200/PO.16.00019 (2017).

66 Javle M., e. a. Pertuzumab + trastuzumab for HER2-positive metastatic biliary cancer: Preliminary data from MyPathway. *Journal of Clinical Oncology* **35**, suppl 402-402, doi:10.1200/JCO.2017.35.4_suppl.402 (2017).

67 Natsumeda, M. *et al.* Detection of 2-Hydroxyglutarate by 3.0-Tesla Magnetic Resonance Spectroscopy in Gliomas with Rare IDH Mutations: Making Sense of "False-Positive" Cases. *Diagnostics (Basel)* **11**, doi:10.3390/diagnostics11112129 (2021).

68 Borger, D. R. *et al.* Frequent mutation of isocitrate dehydrogenase (IDH)1 and IDH2 in cholangiocarcinoma identified through broad-based tumor genotyping. *Oncologist* **17**, 72-79, doi:10.1634/theoncologist.2011-0386 (2012).

69 Shen, X. *et al.* A Noninvasive Comparison Study between Human Gliomas with IDH1 and IDH2 Mutations by MR Spectroscopy. *Metabolites* **9**, doi:10.3390/metabo9020035 (2019).

70 Tarcic, G. e. a. Functional characterization of variants of unknown significance (VUS) in patients and their responsiveness to targeted therapy drugs (TTD). *Annals of Oncology* **27**, VI403, doi:10.1093/annonc/mdw380.09 (2016).

71 Van Cutsem, E. *et al.* Fluorouracil, leucovorin, and irinotecan plus cetuximab treatment and RAS mutations in colorectal cancer. *J Clin Oncol* **33**, 692-700, doi:10.1200/JCO.2014.59.4812 (2015).

72 Douillard, J. Y. *et al.* Panitumumab-FOLFOX4 treatment and RAS mutations in colorectal cancer. *N Engl J Med* **369**, 1023-1034, doi:10.1056/NEJMoa1305275 (2013).

73 Massarelli, E. *et al.* KRAS mutation is an important predictor of resistance to therapy with epidermal growth factor receptor tyrosine kinase inhibitors in non-small-cell lung cancer. *Clin Cancer Res* **13**, 2890-2896, doi:10.1158/1078-0432.CCR-06-3043 (2007).

74 Miller, V. A. *et al.* Molecular characteristics of bronchioloalveolar carcinoma and adenocarcinoma, bronchioloalveolar carcinoma subtype, predict response to erlotinib. *J Clin Oncol* **26**, 1472-1478, doi:10.1200/JCO.2007.13.0062 (2008).

75 Roberts, P. J. & Stinchcombe, T. E. KRAS mutation: should we test for it, and does it matter? *J Clin Oncol* **31**, 1112-1121, doi:10.1200/JCO.2012.43.0454 (2013).

References for Supplementary Tables

76 Linardou, H. *et al.* Assessment of somatic k-RAS mutations as a mechanism associated with resistance to EGFR-targeted agents: a systematic review and meta-analysis of studies in advanced non-small-cell lung cancer and metastatic colorectal cancer. *Lancet Oncol* **9**, 962-972, doi:10.1016/S1470-2045(08)70206-7 (2008).

77 Pao, W. *et al.* KRAS mutations and primary resistance of lung adenocarcinomas to gefitinib or erlotinib. *PLoS Med* **2**, e17, doi:10.1371/journal.pmed.0020017 (2005).

78 Di Donato, N. *et al.* Identification and Characterization of a Novel Constitutional PIK3CA Mutation in a Child Lacking the Typical Segmental Overgrowth of "PIK3CA-Related Overgrowth Spectrum". *Hum Mutat* **37**, 242-245, doi:10.1002/humu.22933 (2016).

79 Dogruluk, T. *et al.* Identification of Variant-Specific Functions of PIK3CA by Rapid Phenotyping of Rare Mutations. *Cancer Res* **75**, 5341-5354, doi:10.1158/0008-5472.CAN-15-1654 (2015).

80 Liu, J., Kimura, A., Baumann, C. A. & Saltiel, A. R. APS facilitates c-Cbl tyrosine phosphorylation and GLUT4 translocation in response to insulin in 3T3-L1 adipocytes. *Mol Cell Biol* **22**, 3599-3609, doi:10.1128/MCB.22.11.3599-3609.2002 (2002).

81 Katzav, S. & Schmitz, M. L. Mutations of c-Cbl in myeloid malignancies. *Oncotarget* **6**, 10689-10696, doi:10.18632/oncotarget.3986 (2015).

82 Levkowitz, G. *et al.* Ubiquitin ligase activity and tyrosine phosphorylation underlie suppression of growth factor signaling by c-Cbl/Sli-1. *Mol Cell* **4**, 1029-1040, doi:10.1016/s1097-2765(00)80231-2 (1999).

83 Han, S. Y. *et al.* Functional evaluation of PTEN missense mutations using in vitro phosphoinositide phosphatase assay. *Cancer Res* **60**, 3147-3151 (2000).

84 Hopman, S. M. *et al.* PTEN hamartoma tumor syndrome and Gorham-Stout phenomenon. *Am J Med Genet A* **158A**, 1719-1723, doi:10.1002/ajmg.a.35406 (2012).

85 Wiencke, J. K. *et al.* Methylation of the PTEN promoter defines low-grade gliomas and secondary glioblastoma. *Neuro Oncol* **9**, 271-279, doi:10.1215/15228517-2007-003 (2007).

86 Rodriguez-Escudero, I. *et al.* A comprehensive functional analysis of PTEN mutations: implications in tumor- and autism-related syndromes. *Hum Mol Genet* **20**, 4132-4142, doi:10.1093/hmg/ddr337 (2011).

87 Lobo, G. P. *et al.* Germline and somatic cancer-associated mutations in the ATP-binding motifs of PTEN influence its subcellular localization and tumor suppressive function. *Hum Mol Genet* **18**, 2851-2862, doi:10.1093/hmg/ddp220 (2009).

References for Supplementary Tables

88 He, X., Ni, Y., Wang, Y., Romigh, T. & Eng, C. Naturally occurring germline and tumor-associated mutations within the ATP-binding motifs of PTEN lead to oxidative damage of DNA associated with decreased nuclear p53. *Hum Mol Genet* **20**, 80-89, doi:10.1093/hmg/ddq434 (2011).

89 He, X. *et al.* Cowden syndrome-related mutations in PTEN associate with enhanced proteasome activity. *Cancer Res* **73**, 3029-3040, doi:10.1158/0008-5472.CAN-12-3811 (2013).

90 Tredan, O. *et al.* Predicting everolimus treatment efficacy in patients with advanced endometrial carcinoma: a GINECO group study. *Target Oncol* **8**, 243-251, doi:10.1007/s11523-012-0242-9 (2013).

91 Yoo, L. I., Chung, D. C. & Yuan, J. LKB1--a master tumour suppressor of the small intestine and beyond. *Nat Rev Cancer* **2**, 529-535, doi:10.1038/nrc843 (2002).

92 Resta, N. *et al.* STK11 mutations in Peutz-Jeghers syndrome and sporadic colon cancer. *Cancer Res* **58**, 4799-4801 (1998).

93 Momcilovic, M. & Shackelford, D. B. Targeting LKB1 in cancer - exposing and exploiting vulnerabilities. *Br J Cancer* **113**, 574-584, doi:10.1038/bjc.2015.261 (2015).
